# Association of LncRNA-PAX8-AS1 and LAIR-2 polymorphisms along with their expression with clinical and subclinical hypothyroidism

**DOI:** 10.1038/s41598-022-26346-0

**Published:** 2023-01-02

**Authors:** Omar M. Elsayed, Samy A. Abdelazim, Hebatallah A. Darwish, Olfat G. Shaker, Mahmoud A. Senousy

**Affiliations:** 1 Egyptian Drug Authority, Cairo, Egypt; 2 Biochemistry Department, Faculty of Pharmacy, Cairo University, Cairo, 11562 Egypt; 3 Pharmacology, Toxicology and Biochemistry Department, Faculty of Pharmacy, Future University in Egypt (FUE), Cairo, Egypt; 4 Medical Biochemistry and Molecular Biology Department, Faculty of Medicine, Cairo University, Cairo, Egypt; 5 Department of Biochemistry, Faculty of Pharmacy and Drug Technology, Egyptian Chinese University, Cairo, 11786 Egypt

**Keywords:** Biochemistry, Genetics, Biomarkers, Diseases, Endocrinology, Molecular medicine

## Abstract

The genetic and epigenetic architecture of clinical and subclinical hypothyroidism remains unclear. We investigated the impact of *long noncoding RNA (LncRNA)-PAX8-AS1* and *LAIR-2* genetic variants on the susceptibility to clinical and subclinical hypothyroidism, their influence on LncRNA-PAX8-AS1 and LAIR-2 expression and their potential as hypothyroid biomarkers. Hundred clinical hypothyroid patients, 110 subclinical hypothyroid patients, and 95 healthy controls were enrolled. Gene expression analysis and genotyping were performed by qPCR. LAIR-2 protein, a proinflammatory mediator, was tested by ELISA. Serum LncRNA-PAX8-AS1 was downregulated, whereas LAIR-2 mRNA and protein levels were upregulated in clinical and subclinical hypothyroid patients compared to healthy controls. *LncRNA-PAX8-AS1* rs4848320 and rs1110839 were associated with increased risk of clinical hypothyroidism. Interestingly, both SNPs were associated with differential expression of serum LncRNA-PAX8-AS1 among clinical hypothyroid patients*. LAIR-2* rs2287828 was associated with elevated risk of both clinical and subclinical hypothyroidism. Harboring the rs2287828 T allele augmented the LAIR-2 mRNA expression among clinical hypothyroid patients, while elevated both LAIR-2 mRNA and protein levels in subclinical hypothyroid patients. The rs4848320-rs1110839-rs2287828 TTT, CTT, and CGT haplotypes were associated with increased hypothyroid risk. Surprisingly, serum LncRNA-PAX8-AS1 and LAIR-2 mRNA expression demonstrated superior diagnostic accuracy for clinical hypothyroidism and turned out as independent predictors in the multivariate analysis. Conclusively, *LncRNA-PAX8-AS1* and *LAIR-2* genetic variants are novel genetic biomarkers of hypothyroidism that could alter the LncRNA-PAX8-AS1 and LAIR-2 expression. LncRNA-PAX8-AS1 and LAIR-2 expression profiles have the potential as effective diagnostic and prognostic indicators of hypothyroidism.

## Introduction

Hypothyroidism is a common condition with devastating health consequences that affect about 5% of the general population. The declined thyroid hormones secretion evokes a decrease in the metabolic rate and can incur a life-threatening myxedema and other symptoms resulting in a great social and economic encumbrance^1,2^.

According to the degree of thyroid function reduction, clinical and subclinical hypothyroidism cases were distinguished^1,2^. Subclinical hypothyroidism, the earliest biochemical abnormality in hypothyroidism, is characterized by mildly elevated serum thyrotropin or thyroid stimulating hormone (TSH) levels recording > 4.5 mIU/L but < 10 mIU/L, associated with normal serum thyroxine (T4) and triiodothyronine (T3) concentrations in the absence of obvious clinical manifestations. While clinical hypothyroidism, the latest biochemical abnormality in hypothyroidism, is recognized by higher levels of TSH (> 10 mIU/L), associated with a decline in both serum T3 and T4 levels, at which stage most patients have obvious clinical symptoms (overt hypothyroidism)^3,4^.

Interestingly, the prevalence of subclinical hypothyroidism is substantially higher than that of overt hypothyroidism (4 to 10% versus 0.1 to 2%, respectively) in the general population. When non-age-specific TSH reference values are employed, up to 15% of persons 65 years or older have subclinical hypothyroidism. Women are nearly ten times more likely than men to develop spontaneous hypothyroidism^4,5^. Despite such high incidence, the pathogenesis of subclinical hypothyroidism remains elusive. Thereby, decoding the molecular underpinnings of thyroid pathology could identify new potential targets for early detection and treatment of subclinical hypothyroidism, and hence slowing down the progression to clinical hypothyroidism.

Besides the well-known causative factors of hypothyroidism–iodine deficiency and autoimmunity^5^-genetic, epigenetic, and environmental factors are implicated in its development by altering the thyroid function^6,7^; however, studies on such genetic and epigenetic molecules are still lacking.

Long noncoding RNAs (lncRNAs, longer than 200 nucleotides in length) fine-tune gene expression by recruiting epigenetic complexes or directly affecting transcription of multiple genes acting on various cellular paradigms^8,9^. The expression of lncRNAs is exceedingly controlled, with organ, tissue and even cellular specific patterns^8,10^ and they have been documented to have crucial roles in the normal development, function, physiology, and pathophysiology of several endocrine organs such as the prostate, pancreas, and adipose tissue^11,12^. However, little is known about the biological function of lncRNAs and their expression pattern in the thyroid gland.

Importantly, several molecules were deduced to function in hypothyroidism, the lncRNA-paired-box gene 8-antisense RNA 1 (LncRNA-PAX8-AS1) and the leukocyte-associated Ig-like receptor-2 (LAIR-2) were proposed as one of the most important in such condition^1,13^.

The *LncRNA-PAX8-AS1*, mapped to chromosome 2q13 upstream of *PAX8* gene, is a potential regulator of PAX8^14^. PAX8 was regarded as a nuclear transcription factor of the mammalian PAX protein family in the developing and adult thyroid^15,16^. Interestingly, PAX8 is the master regulator of the differentiated thyroid phenotype and is required in thyroid cells for transcriptional activation of thyroglobulin, thyroid peroxidase, and sodium iodide symporter^16^.

An expression quantitative trait locus (eQTL) is a locus containing a genetic variant that impact the expression level of a gene^17,18^. The *LncRNA-PAX8-AS1* gene locus has been shown to encompass polymorphisms that represent eQTLs for PAX8. Specifically, the single nucleotide polymorphisms (SNPs) rs4848320 and rs1110839 in the *LncRNA-PAX8-AS1* gene are candidate eQTLs of PAX8 as revealed by previous bioinformatics analyses. These studies raised the possibility that these variants might influence the expression, function or structure of the LncRNA-PAX8-AS1, thereby amending the expression of PAX8^14,19^. However, the mechanistic role of these SNPs and whether they govern the expression of LncRNA-PAX8-AS1 in hypothyroidism are not previously explored.

The leukocyte-associated Ig-like receptor (LAIR) is a small family of immune receptor tyrosine-based inhibitory motifs (ITIM)-containing inhibitory receptor that belongs to the Ig superfamily^20^. *LAIR-2* gene is located on human chromosome 19q13.4. It encodes LAIR-2 protein, a secreted receptor acting as a pro-inflammatory mediator through limiting the potential of the immune inhibitor LAIR-1*.* This results in enhanced activation of immune cells, the hallmark of autoimmune hypothyroid disease^13,21^. Importantly, LAIR molecules interact with collagens with high affinity. Peculiarly, the interaction of LAIR-1 with collagens directly inhibited immunocyte activation in vitro, suggesting a vital mechanism of peripheral immune regulation through the extracellular matrix^21^. LAIR-2 plays a significant role in such regulation based on its higher affinity for collagens than LAIR-1. Indeed, elevated levels of serum LAIR-2 exhibited a potential regulatory capability in modulating the inhibitory power of LAIR-1 in patients with autoimmune thyroid diseases^13^.

It has been found that some SNPs located near the *LAIR-2* gene may influence the susceptibility to autoimmune diseases. For instance, the *LAIR-2* SNP rs2287828 has been shown to be associated with the susceptibility to ankylosing spondylitis and pemphigus diseases and was found to affect the LAIR-2 mRNA expression level^22,23^. However, the impact of this SNP on the risk of hypothyroid disease and its subclinical condition as well as its possible control on LAIR-2 mRNA and protein expression in hypothyroidism remains to be investigated.

Therefore, this study aimed to investigate the possible role of *LncRNA-PAX8-AS1* and *LAIR-2* genetic variants in the development of both subclinical and clinical (overt) hypothyroidism. The study also explored the expression pattern of LncRNA-PAX8-AS1, LAIR-2 mRNA and protein, their diagnostic and prognostic potential, and their correlations with clinical data in both conditions. The mechanistic influence of the studied SNPs on gene expression and their association with patients’ parameters were also assessed.

## Results

### Demographic and clinical characteristics of the studied groups

As shown in Table 1, both hypothyroid groups have the same age and gender distribution. Comparing with healthy controls, hypothyroid patients exhibited significantly higher body mass index (BMI), thyroid volume, serum TSH, total cholesterol, LDL-cholesterol, triglycerides, fasting plasma glucose, 2-h postprandial (2HPP) plasma glucose, blood glycated hemoglobin (HbA1c) and fasting plasma insulin, along with significantly lower serum HDL-cholesterol levels (*P* < 0.05). However, clinical hypothyroid patients exhibited significantly higher thyroid volume, serum TSH, triglycerides, fasting plasma glucose, 2HPP blood glucose, HbA1c and fasting plasma insulin levels when compared with levels in the subclinical hypothyroid patients (*P* < 0.05). On the other hand, only clinical hypothyroid patients exhibited significantly lower serum levels of free T4 and free T3 than those in healthy controls (*P* < 0.05), whereas subclinical hypothyroid patients did not show any significant difference regarding free T4 and T3 levels when compared with the healthy controls levels (*P* > 0.05).

**Table 1:** Demographic and clinical characteristics of the studied groups.

Parameters	Groups			*P* value
	Healthy controls (n = 95)	Hypothyroid patients		
		Subclinical (n = 110)	Clinical (n = 100)	
Age (years)	36.63 ± 8.76	37.64 ± 7.74	39.15 ± 8.98	0.113
Gender				0.67
Female, n (%)	87 (91.58%)	104 (94.55%)	92 (92%)	
Male, n (%)	8 (8.42%)	6 (5.45%)	8 (8%)	
BMI (kg/m^2^)	22.57 ± 2.94	29.7 ± 6.22^#^	30.51 ± 4.96^#^	** < 0.0001**
TSH (mIU/L)	1.57 (1.16–2.02)	7.68(6.07–12.2)^#^	26.35 (14.25–75.38)^#$^	** < 0.0001**
Free T4 (ng/dL)	1.26 ± 0.12	1.33 ± 0.5	0.61 ± 0.22^#$^	** < 0.0001**
Free T3 (pg/mL)	3 ± 0.53	3.11 ± 0.59	2.01 ± 0.69^#$^	** < 0.0001**
Thyroid volume (mL)	2.69 (2.14–3.94)	4.35 (2.64–6.65)^#^	7.1 (4.76–8.52)^#$^	** < 0.0001**
**Lipid profile**				
Total cholesterol (mg/dL)	159.4 ± 28.72	181.8 ± 45.6^#^	196.7 ± 47.80^#^	** < 0.0001**
LDL-cholesterol (mg/dL)	91.81 ± 28.02	111.1 ± 40.05^#^	125.3 ± 44.94^#^	** < 0.0001**
HDL-cholesterol (mg/dL)	55.17 ± 14.41	46.9 ± 12.59^#^	43.81 ± 9.71^#^	** < 0.0001**
Triglycerides (mg/dL)	64 (47–96)	105 (60.8–156.5)^#^	132 (93.25–172.3)^#$^	** < 0.0001**
**Sugar profile**				
Fasting plasma glucose (mg/dL)	71.8 ± 3.9	80.71 ± 9.22^#^	85.69 ± 8.87^#$^	** < 0.0001**
2HPP blood glucose (mg/dL)	100 ± 6.6	116 ± 15.18^#^	126.4 ± 10.87^#$^	** < 0.0001**
HbA1c (%)	4.69 ± 0.27	4.98 ± 0.3^#^	5.37 ± 0.36^#$^	** < 0.0001**
Fasting plasma insulin (IU/L)	5.2 (4.3–11.5)	8.3 (4.8–14.03)^#^	9.35 (6.8–16.58)^#$^	** < 0.0001**

Data are presented as mean ± SD, median (25–75% percentiles) or number (percentage). BMI: body mass index, HbAIc: glycated hemoglobin, 2HPP: 2-h postprandial, TSH: thyroid stimulating hormone. ^#^Statistical significance from healthy controls, ^$^Statistical significance from subclinical hypothyroid patients. *P* values of ANOVA or Kruskal Wallis tests (when appropriate) across the three groups are presented. Bold means statistical significance between groups, *P* < 0.05.

### Association of rs4848320 (C/T), rs1110839 (G/T) and rs2287828 (C/T) with the risk of clinical and subclinical hypothyroidism

For all studied SNPs, the minor allele frequency (MAF) in the control group was > 0.05 (Table 2). In the study controls, MAF was slightly higher than the reported global MAF (T = 0.21) for rs4848320 and slightly lower than the global MAF (T = 0.48 and T = 0.11) for rs1110839 and rs2287828, respectively (Table 2). However, it was close to that reported for some populations for the three SNPs as recorded in the Ensembl GRCh38 release 102-November 2020. As displayed in Supplementary Table S1, the distribution of the rs4848320, rs1110839, and rs2287828 genotypes in the study groups did not deviate from HWE (*P* > 0.05).

**Table 2:** Basic information of studied single nucleotide polymorphisms and odds ratio estimates for subclinical and clinical hypothyroidism.

gene name	SNP ID (allele)	Global MAF* (T allele)	MAF (healthy controls)	MAF (subclinical hypothyroidism)	OR^a^ (95% CI)	*P*^a^ value	MAF (clinical hypothyroidism)	OR^a^ (95% CI)	*P*^a^ value
*LncRNA-PAX8-AS1*	rs4848320 (C/T)	T = 0.21	T = 0.26	T = 0.31	1.28 (0.82–1.99)	0.27	T = 0.42	2.01 (1.30–3.09)	**0.0015**
	rs1110839 (G/T)	T = 0.48	T = 0.3	T = 0.31	1.03 (0.68–1.58)	0.88	T = 0.495	2.22 (1.46–3.38)	** < 0.0001**
*LAIR-2*	rs2287828 (C/T)	T = 0.11	T = 0.08	T = 0.36	6.64 (3.65–12.06)	** < 0.0001**	T = 0.43	8.50 (4.66–15.49)	** < 0.0001**

*MAF* minor allele frequency. *According to Ensembl release 102—November 2020. *OR* odds ratio, *CI* confidence interval. ^a^Adjusted for age and sex in a logistic regression model using SNPstats online software. *P* values in bold means statistical significance from healthy controls (*P* < 0.05).

The best fit genetic models of rs4848320, rs1110839, and rs2287828 in clinical and subclinical hypothyroid patients against controls are demonstrated in Table 3. For rs4848320 (C/T) and rs1110839 (G/T) in the *LncRNA-PAX8-AS1* gene, the minor T allele was associated with increased risk of clinical hypothyroidism (adjusted OR = 2.01, *P* = 0.0015 and 2.2, *P* < 0.0001, respectively) with adjustment for age and sex as confounding factors (Table 2). In clinical hypothyroid patients, the rs4848320 and rs1110839 log-additive models were significant and showed a noticeable clinical hypothyroid risk (adjusted OR = 2.26 and 2.55, respectively, *P* < 0.0001). In addition, among the subclinical hypothyroid patients, the genotype distribution and allele frequencies of rs4848320 and rs1110839 did not show a statistically significant difference when compared with those in the healthy controls (*P* > 0.05) (Table 3).

**Table 3:** Association of *LncRNA-PAX8-AS1* rs4848320 (C/T), rs1110839 (G/T), and *LAIR-2* rs2287828 (C/T) with subclinical and clinical hypothyroidism.

SNP ID (allele)	Model	Allele or genotype	Healthy controls (n = 95)	Subclinical hypothyroidism (n = 110)	OR^a^ (95% CI)	*P*^a^ value	AIC	BIC
rs4848320 (C/T)	Allelic*	C	140 (0.74)	152 (0.69)	r			
		T	50 (0.26)	68 (0.31)	1.28 (0.82–1.99)	0.27	285.1	293.1
rs1110839 (G/T)	Allelic*	G	132 (0.7)	151 (0.69)	r			
		T	58 (0.3)	69 (0.31)	1.03 (0.68–1.58)	0.88	285.7	293.7
rs2287828 (C/T)	Dominant*	CC	82 (86.3%)	47 (42.7%)	r			
		CT + TT	13 (13.7%)	63 (57.3%)	8.70 (4.31–17.59)	** < 0.0001**	244.8	258.1
SNP ID (allele)	Model	Genotype	Healthy controls (n = 95)	Clinical hypothyroidism (n = 100)	OR^a^ (95% CI)	*P*^a^ value	AIC	BIC
rs4848320 (C/T)	Log additive*	–	–	–	2.26 (1.39–3.68)	** < 0.0001**	261.9	271.5
rs1110839 (G/T)	Log additive*	–	–	–	2.55 (1.58–4.11)	** < 0.0001**	257.4	270.3
rs2287828 (C/T)	Dominant*	CC	82 (86.3%)	30 (30%)	r			
		CT + TT	13 (13.7%)	70 (70%)	14.51 (6.98–30.17)	** < 0.0001**	221.8	208.7

Values are expressed as number (percentage). ^a^adjusted for age and sex in a logistic regression model using SNPstats online software. * refers to the model that best matches the data and was chosen using the lowest Akaike information criterion (AIC) and Bayesian information criterion (BIC) values compared to other genetic models. *P* values in bold means statistical significance, *P* < 0.05.

Interestingly, the minor T allele frequency of *LAIR-2* rs2287828 (C/T) was associated with 8.50 and 6.64-fold increase in the risk of clinical and subclinical hypothyroidism, respectively (T vs. C allele, *P* < 0.0001 for each) as shown in Table 2. Moreover, a markedly high risk for clinical and subclinical hypothyroidism was also depicted from the rs2287828 dominant (CT + TT vs CC) model (Adjusted OR = 14.51 and 8.7, respectively, *P* < 0.0001) (Table 3). Like the other two SNPs, all results were adjusted for age and sex as covariates.

### Stratification analysis

The effect of rs4848320 (C/T), rs1110839 (G/T), and rs2287828 (C/T) SNPs on clinical and subclinical hypothyroid risk were further examined in a stratified risk analysis by gender (Supplementary Table S2). Among female patients, rs4848320 (C/T) and rs1110839 (G/T) log additive models revealed high risk for clinical hypothyroidism (adjusted OR = 2.94 and 2.51, respectively, *P* < 0.0001). Moreover, rs2287828 dominant model (CT + TT vs CC) showed a markedly elevated susceptibility to clinical and subclinical hypothyroidism among the female patients (adjusted OR = 21.61 and 12.77, respectively, *P* < 0.0001). Noticeably, stratified analysis among the male patients did not show any susceptibility risk (*P* > 0.05).

### Haplotype analysis

The combined impact of the studied gene polymorphisms in all hypothyroid patients compared with healthy controls was evaluated (Table 4). Results revealed that the rs4848320-rs1110839-rs2287828 TTT, CTT, and CGT haplotypes were associated with increased risk of hypothyroidism by 10.15, 27.45 and 9.42-fold, respectively (TTT vs CGC, *P* < 0.0001, CTT vs CGC, *P* = 0.038, and CGT vs CGC, *P* = 0.027). Other haplotypes were not statistically associated with the risk of hypothyroidism (*P* ≥ 0.05).

**Table 4:** Association of haplotypes with hypothyroid risk.

Haplotype			Total frequency	Frequency in healthy controls	Frequency in all hypothyroid groups	OR^a^ (95% CI)	*P*^a^ value
*LncRNA-PAX8-AS1* rs4848320	*LncRNA-PAX8-AS1* rs1110839	*LAIR-2* rs2287828					
C	G	C	0.3893	0.5203	0.3287	1	–
C	T	C	0.1462	0.1978	0.123	0.95 (0.50–1.82)	0.88
T	G	C	0.1247	0.0226	0.1258	1.70 (0.82–3.54)	0.16
T	T	T	0.1055	0.1267	0.1431	10.15 (2.74–37.65)	** < 0.0001**
C	T	T	0.074	0.0376	0.1042	27.45 (1.21–621.48)	**0.038**
C	G	T	0.0593	0.0762	0.0821	9.42 (1.30–68.08)	**0.027**
T	G	T	0.0562	0.0101	0.0634	2.97 (1.00–8.80)	0.05
T	T	C	0.0447	0.0087	0.0296	0.62 (0.21–1.85)	0.39

Healthy controls, n = 95, all hypothyroid patients, n = 210. ^a^adjusted by age and sex in a logistic regression model using SNPstats online software. Global haplotype association manifested at *P* < 0.0001. T allele is the risk one for all SNPs. *P* values in bold means statistical significance between groups (*P* < 0.05).

### Serum LncRNA-PAX8-AS1 and LAIR-2 mRNA expression levels in clinical and subclinical hypothyroidism

**Figure 1:**
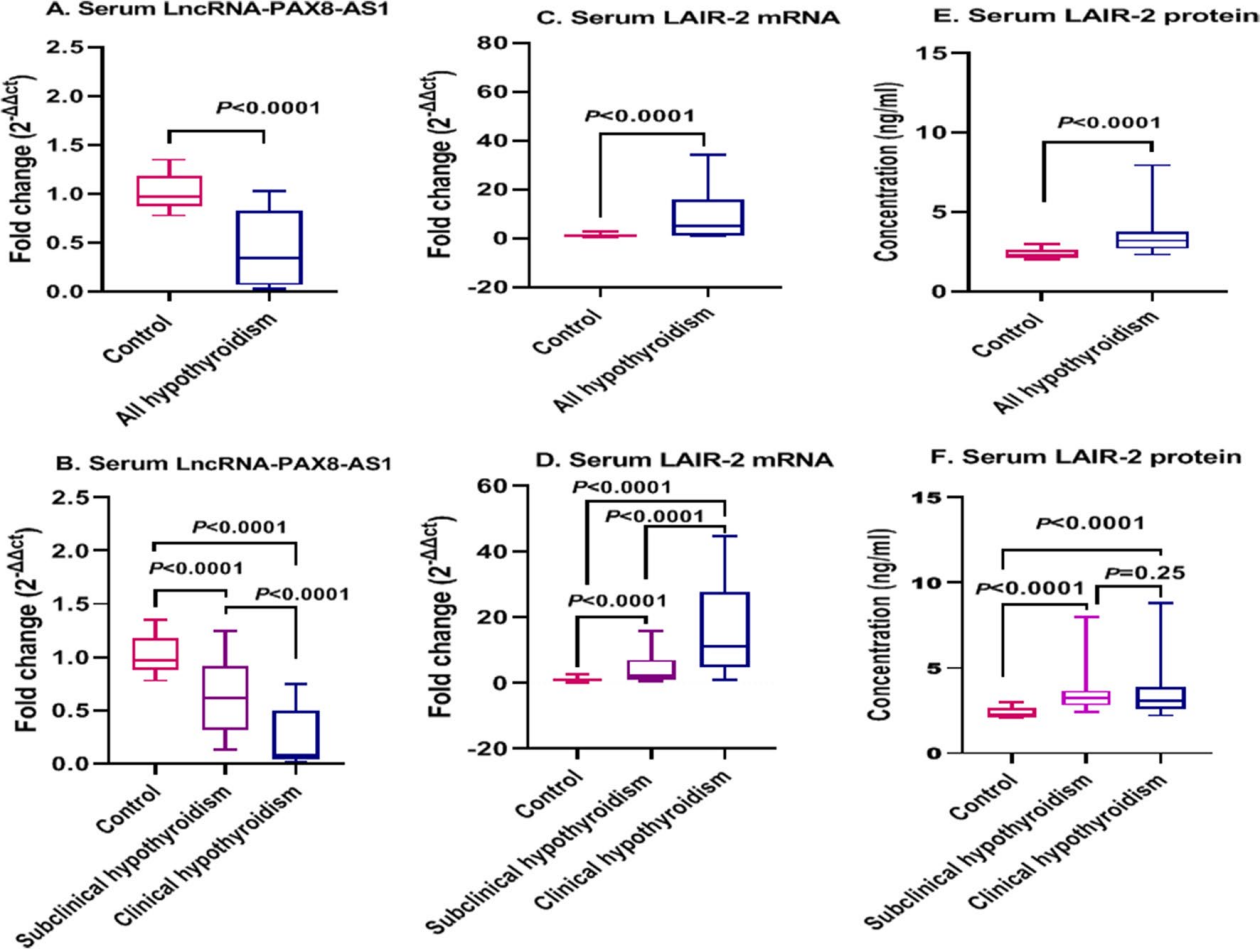
Serum expression levels of LncRNA-PAX8-AS1 and LAIR-2 mRNA and protein in the studied groups. (**A)**, (**C)**, (**E)** all hypothyroidism patients (n = 210) versuss healthy controls (n = 95). (**B**) (**D**), (**F**) Clinical hypothyroid patients (n = 100) and subclinical hypothyroid patients (n = 110) versus healthy controls (n = 95). Data are expressed as box blot; the box represents the 25–75% percentiles; the line inside the box represents the median and the upper and lower lines representing the 10–90% percentiles. *P* < 0.05 means statistical significance.

As depicted in Fig. 1A, the expression levels of serum lncRNA-PAX8-AS1 were markedly lower in all hypothyroid groups than those in the healthy controls (*P* < 0.0001). In-depth analysis showed that serum LncRNA-PAX8-AS1 expression was markedly downregulated with a median fold change = 0.073 and 0.611 (*P* < 0.0001 for each) in clinical and subclinical hypothyroid patients, respectively compared with that in the healthy controls, with serum lncRNA-PAX8-AS1 recorded significantly lower levels in clinical hypothyroid patients than those in the subclinical hypothyroid patients (*P* < 0.0001) (Fig. 1B).

Referring to Fig. 1C, serum LAIR-2 mRNA expression levels were significantly higher in all hypothyroid groups than those in the healthy controls (*P* < 0.0001). The detailed results (Fig. 1D) showed that serum LAIR-2 mRNA expression was upregulated with a median fold change of 11.29 and 2.11 (*P* < 0.0001 for each) in clinical and subclinical hypothyroid patients, respectively, compared with that in the healthy controls. Also, clinical hypothyroid patients demonstrated significantly higher LAIR-2 mRNA levels than those in the subclinical hypothyroid patients (*P* < 0.0001).

### Serum LAIR-2 protein levels in clinical and subclinical hypothyroidism

Serum LAIR-2 protein levels were significantly higher in all hypothyroid groups than the levels in the healthy controls (*P* < 0.0001) (Fig. 1E). Interestingly, LAIR-2 protein revealed higher serum levels in clinical and subclinical hypothyroid groups with a median of 3.09 and 3.24 than those in the healthy controls, respectively (*P* < 0.0001 for each), nevertheless, there was no significant difference between clinical and subclinical hypothyroid groups (*P* = 0.25) (Fig. 1F).

### Association of rs4848320, rs1110839, and rs2287828 SNPs with serum LncRNA-PAX8-AS1, LAIR-2 mRNA, and LAIR-2 protein levels in clinical and subclinical hypothyroid patients

**Figure 2:**
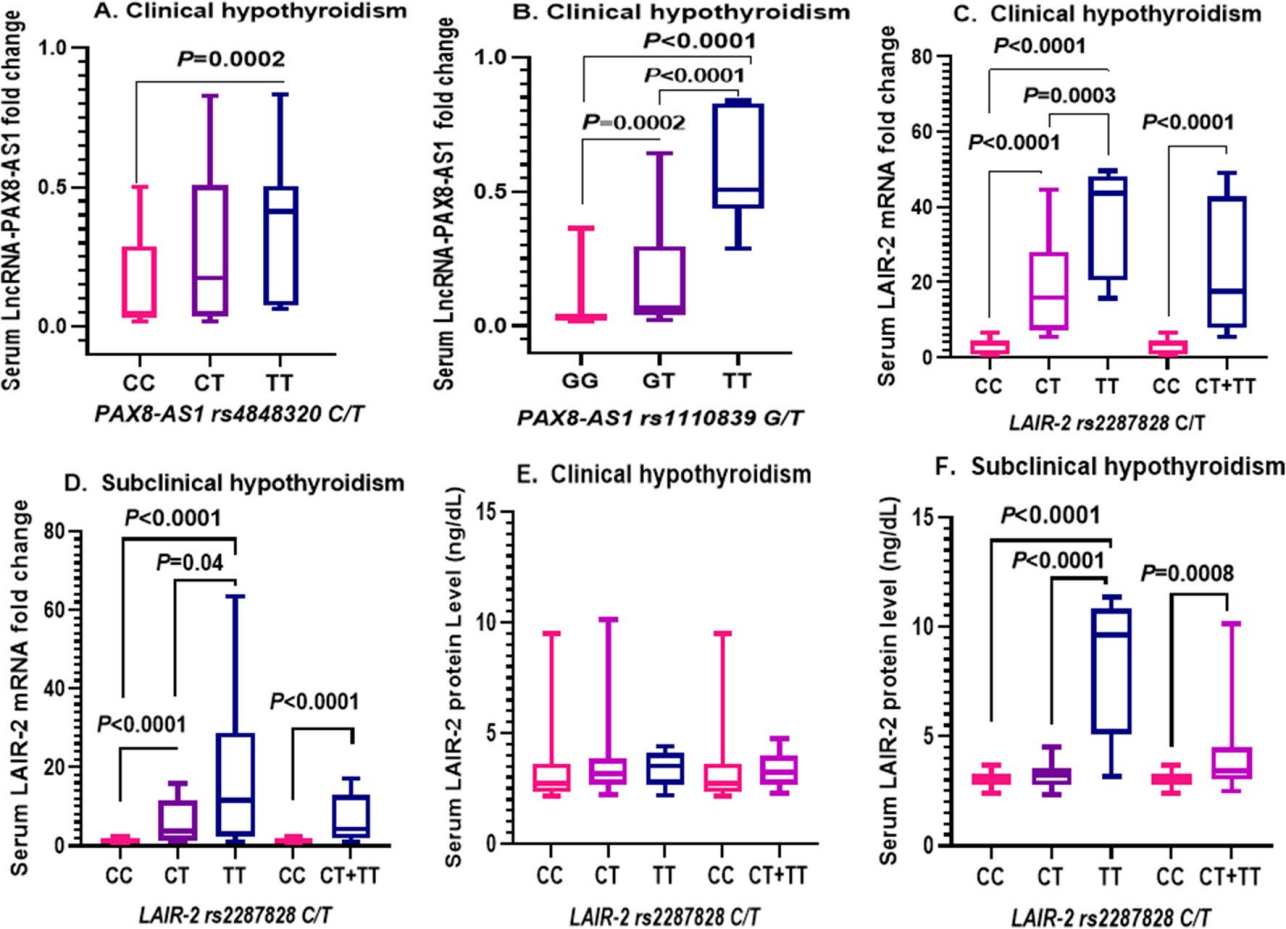
Influence of studied SNPs on the gene expression of LncRNA-PAX8-AS1, LAIR-2 mRNA and protein in hypothyroidism patients. (**A**, **B**) Fold change of serum expression levels of LncRNA-PAX8-AS1 in different *LncRNA-PAX8-AS1* rs4848320 genotypes (CC, n = 30, CT, n = 56, TT, n = 14) and rs1110839 genotypes (GG, n = 22, GT, n = 57, TT, n = 21) in clinical hypothyroidism patients, respectively. (**C**, **D**) Fold change of serum expression of LAIR-2 mRNA, (**E**, **F**) Serum LAIR-2 protein concentrations in different *LAIR-2* rs2287828 genotypes in clinical hypothyroidism (CC, n = 30, CT, n = 54, TT, n = 16) and subclinical hypothyroidism (CC, n = 47, CT, n = 47, TT, n = 16) patients, respectively. Data are expressed as box blot; the box represents the 25–75% percentiles; the line inside the box represents the median and the upper and lower lines representing the 10–90% percentiles. *P* < 0.05 means statistical significance.

Assessment of serum LncRNA-PAX8-AS1, LAIR-2 mRNA, and LAIR-2 protein levels in clinical and subclinical hypothyroid patients carrying different SNP genotypes was performed in order to study the mechanistic role of rs4848320, rs1110839, and rs2287828 in hypothyroidism (Fig. 2). For rs4848320, we found that the serum LncRNA-PAX8-AS1 expression levels were significantly higher in clinical hypothyroid TT genotype carriers than those in the CC genotype carriers (*P* = 0.0002) (Fig. 2A). Regarding rs1110839, serum LncRNA-PAX8-AS1 expression levels were also markedly higher in the clinical hypothyroid TT genotype carriers than those in the GG or GT genotype carriers (*P* < 0.0001) and in the GT genotype carriers than those in the GG carriers (*P* = 0.0002) (Fig. 2B).

Regarding rs2287828, serum LAIR-2 mRNA expression levels were significantly higher in the clinical or subclinical hypothyroid TT genotype carriers than in those carrying the CC or CT genotype as well as in CT vs CC genotype carriers (*P* < 0.05). Also, serum LAIR-2 mRNA expression levels were significantly higher in the CT + TT vs CC comparison (*P* < 0.0001) (Fig. 2C,D).

There was no significant difference found in serum LAIR-2 protein levels among the clinical hypothyroid patients carrying different rs2287828 genotypes (*P* > 0.05) (Fig. 2E). However, serum LAIR-2 protein levels were significantly higher in the subclinical hypothyroid patients harboring the TT genotype than those having the CC or CT genotypes (*P* < 0.0001) and in the CT + TT vs CC comparison as well (*P* = 0.0008) (Fig. 2F).

### Diagnostic performance of LncRNA-PAX8-AS1, LAIR-2 mRNA, and LAIR-2 protein

**Figure 3:**
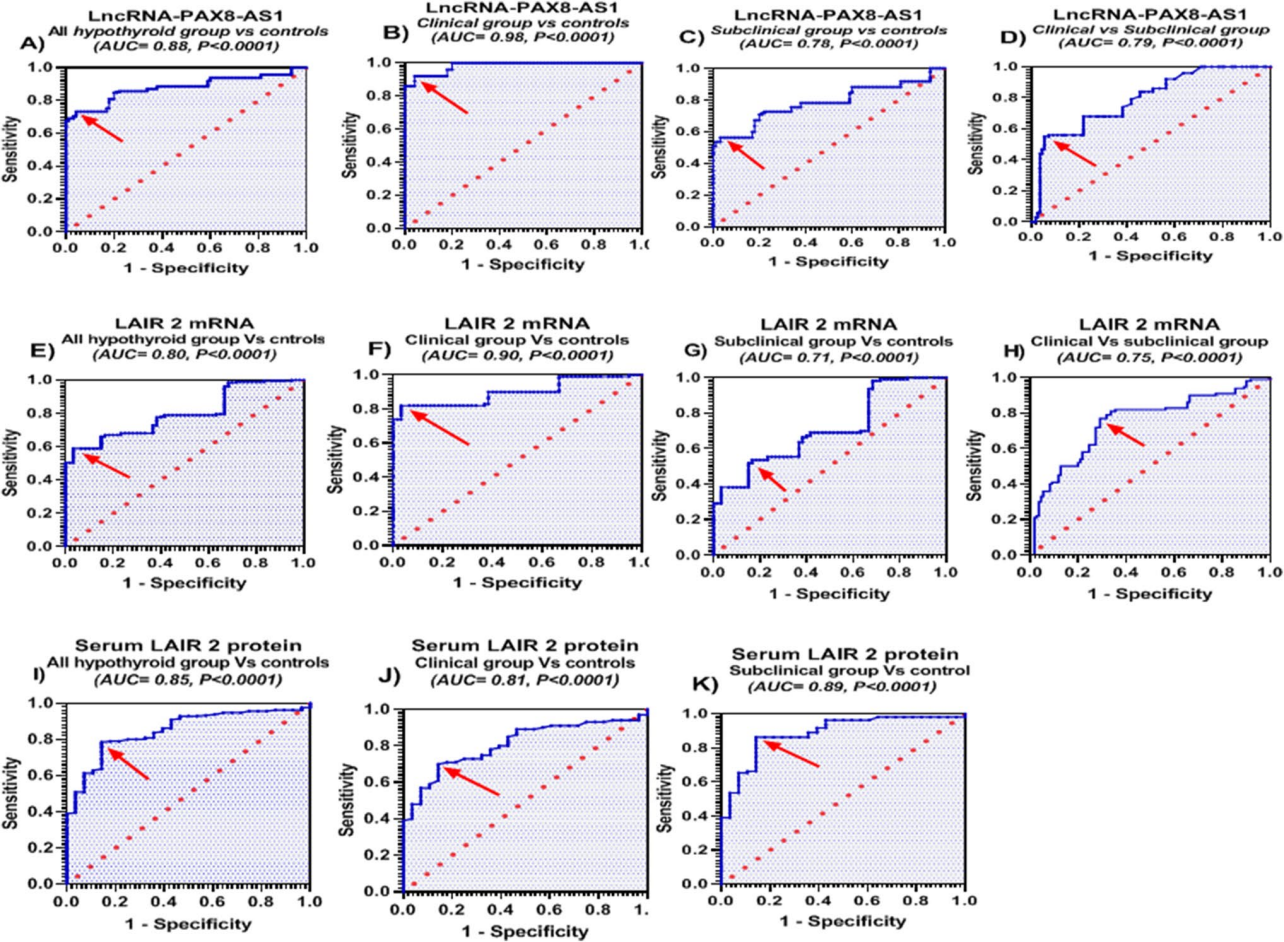
Diagnostic performance of serum lncRNA-PAX8-AS1, LAIR-2 mRNA and protein in studied groups using ROC curve analysis. Clinical hypothyroid group (n = 100), subclinical hypothyroid group (n = 110), all hypothyroid group (n = 210), and healthy controls (n = 95). The arrow points at the best cutoff point. *P* < 0.05 means statistical significance.

Reciever operating characteristic (ROC) curve analysis of the studied groups revealed that serum LncRNA-PAX8-AS1 significantly distinguished all hypothyroid group as well as clinical and subclinical hypothyroid patients from healthy controls with area under the curve (AUC) = 0.88, 0.98, and 0.78, respectively (Fig. 3A–C). In addition, serum LncRNA-PAX8-AS1 discriminated the clinical hypothyroid patients from subclinical hypothyroid group with AUC = 0.79 (Fig. 3D). These data suggest serum LncRNA-PAX8-AS1 as a potential discriminator for hypothyroidism with an excellent diagnostic performance for clinical hypothyroid patients (Table 5).

**Table 5:** Diagnostic accuracy of the studied markers distinguishing candidate groups.

Serum marker	Cutoff	AUC	95% CI	*P* value	Sensitivity (%)	Specificity (%)
**All hypothyroid groups versus healthy controls**						
LncRNA-PAX8-AS1	< 0.75-fold	0.88	0.84–0.916	< 0.0001	73.33	95.79%
LAIR-2 mRNA	> 2.7-fold	0.80	0.745–0.853	< 0.0001	59.05	96.67%
LAIR-2 protein	> 2.7-fold	0.85	0.787–0.913	< 0.0001	78.57	85.71
**Clinical hypothyroid patients versus healthy controls**						
LncRNA-PAX8-AS1	< 0.74-fold	0.98	0.969–0.995	< 0.0001	92	95.79%
LAIR-2 mRNA	> 2.9-fold	0.90	0.849–0.946	< 0.0001	82	96.67%
LAIR-2 protein	> 2.7-fold	0.81	0.731–0.887	< 0.0001	70	85.71%
**Subclinical hypothyroid patients versus healthy controls**						
LncRNA-PAX8-AS1	< 0.73-fold	0.78	0.719–0.848	< 0.0001	56.36	96.84%
LAIR-2 mRNA	> 1.9-fold	0.71	0.631–0.787	< 0.0001	53.64	83.33%
LAIR-2 protein	> 2.6-fold	0.89	0.823–0.952	< 0.0001	86.36	85.71%
**Clinical versus subclinical hypothyroid patients**						
LncRNA-PAX8-AS1	< 0.09-fold	0.79	0.729–0.851	< 0.0001	55	94.55%
LAIR-2 mRNA	> 4.6-fold	0.75	0.682–0.817	< 0.0001	77	70.91%

*AUC* area under the curve, *CI* confidence interval*. P* < 0.05 means statistical significance.

Importantly, the analysis also revealed that serum LAIR-2 mRNA distinguished all hypothyroid group, clinical, and subclinical patients (Fig. 3E–G) from healthy controls with a sufficient performance power (AUC = 0.8, 0.9, and 0.71, respectively) and with an excellent discrimination of clinical hypothyroidism. Moreover, serum LAIR-2 mRNA discriminated the clinical hypothyroid patients from subclinical hypothyroid group with AUC = 0.75 (Fig. 3H). These data highlighted that serum LAIR-2 mRNA expression could be a promising discriminator of hypothyroidism and its subtypes. LAIR-2 protein discriminated all hypothyroid, clinical, and subclinical hypothyroid patients from healthy controls as well with AUC = 0.85, 0.81, and 0.89, respectively (Fig. 3I–K), indicating its diagnostic potential. The calculated sensitivities and specificities at the best cutoff values are shown in Table 5.

### Correlation of LncRNA-PAX8-AS1, LAIR-2 mRNA, and LAIR-2 protein levels with the demographic and clinical data of clinical hypothyroidism

As shown in Supplementary Table S3, no significant correlations were observed for LncRNA-PAX8-AS1 or LAIR-2 mRNA levels with the laboratory data of clinical hypothyroid patients. However, serum LAIR-2 protein levels were positively correlated with total cholesterol (r = 0.21, *P* = 0.04) and LDL-cholesterol concentrations (r = 0.28, *P* = 0.004) in clinical hypothyroid patients.

### Association of rs4848320, rs1110839, and rs2287828 with the demographic and clinical data of clinical hypothyroidism

To explore the correlation of rs4848320, rs1110839, and rs2287828 with clinical hypothyroidism, we assessed the laboratory data in the clinical hypothyroid patients carrying different SNP genotypes (Supplementary Figures S1, S2, and S3). For rs4848320, we found no significant correlations of the different genotypes with the laboratory data of clinical hypothyroid patients (Supplementary Figure S1). Regarding rs1110839, we found that the clinical hypothyroid patients carrying the GG genotype had a significant lower serum free T3 levels than those in the TT genotype carriers (*P* = 0.02) (Supplementary Figure S2-D) and a significant smaller thyroid volume than that in the GT + TT genotype carriers (*P* = 0.045) (Supplementary Figure S2-E). In addition, lipid profile assessment in clinical hypothyroid patients carrying different rs1110839 genotypes revealed higher serum triglyceride levels in the TT genotype carriers than those in the GT (*P* = 0.017) or GG + GT (*P* = 0.026) genotype carriers (Supplementary Figure S2-I). However, sugar profile assessment in clinical hypothyroid patients carrying different rs1110839 genotypes revealed higher fasting plasma glucose levels in those harboring the GG genotype than in those carrying the GT + TT genotype (*P* = 0.04) (Supplementary Figure S2-J). Furthermore, the clinical hypothyroid patients carrying the GG genotype exhibited significantly higher 2HPP plasma glucose, blood HbA1c, and fasting plasma insulin levels than the levels in patients holding the GT or GT + TT genotypes (*P* < 0.05) (Supplementary Figures S2-K, L, and M, respectively).

Regarding rs2287828, we found that the clinical hypothyroid patients carrying the TT genotype exhibited a significantly higher serum total cholesterol levels than those in the CC + CT genotypes carriers (*P* = 0.024) (Supplementary Figure S3-F) and a significantly higher serum triglyceride concentrations than in those harboring the CC or CC + CT genotypes (*P* < 0.05) (Supplementary Figure S3-I). For the glycemic profile, we found that clinical hypothyroid patients carrying the TT genotype had higher serum fasting plasma insulin levels than those having the CC + CT genotype carriers (*P* = 0.047) (Supplementary Figure S3-M).

### Results of logistic regression analysis

The predictor parameters associated with the risk of clinical hypothyroidism among both subclinical hypothyroid group and healthy controls were selected using a univariate followed by a multivariate logistic regression analysis (Table 6). Expression levels of serum LncRNA-PAX8-AS1 and LAIR-2 mRNA were selected as significant predictors associated with the possibilities of clinical hypothyroidism diagnosis in the univariate analysis (*P* < 0.05). In a stepwise forward multivariate analysis, LncRNA-PAX8-AS1 and LAIR-2 mRNA also turned out to be significant negative and positive predictors of clinical hypothyroid risk, respectively, in this study (*P* < 0.05). Additionally, expression levels of LncRNA-PAX8-AS1 and LAIR-2 mRNA as well as its protein turned out as significant final predictors for subclinical hypothyroid risk among healthy controls in the multivariate analysis (*P* < 0.05) (Table 7).

**Table 6:** Logistic regression analysis to predict the risk of clinical hypothyroidism.

Parameter	β-coefficient	SE	*P*^a^ value	OR^a^	OR (95% CI)
**Univariate analysis**					
LncRNA-PAX8-AS1	− 4.126	0.475	** < 0.0001**	0.016	0.006–0.041
LAIR-2 mRNA	0.101	0.017	** < 0.0001**	1.110	1.107–1.114
LAI-2 protein	0.053	0.060	0.379	1.055	0.937–1.187
**Multivariate analysis**					
LncRNA-PAX8-AS1	− 3.646	0.531	** < 0.0001**	0.026	0.009–0.074
LAIR-2 mRNA	0.074	0.015	** < 0.0001**	1.076	1.044–1.110
Constant	0.970				

Clinical hypothyroidism (n = 100) versus healthy control + subclinical hypothyroid group (n = 205). Log likelihood of the stepwise multivariate logistic regression model =  − 107.847, − 2 Log likelihood = 215.694, *P* < 0.0001. *P* values in bold means statistical significance between groups, *P* < 0.05.^a^ adjusted for age and sex.

**Table 7:** Logistic regression analysis to predict the risk of subclinical hypothyroidism.

Parameter	β coefficient	SE	*P*^a^ value	OR^a^	OR (95% CI)
**Univariate analysis**					
LncRNA-PAX8-AS1	− 2.142	0.42	** < 0.0001**	0.177	0.052–0.267
LAIR-2 mRNA	0.442	0.143	**0.002**	1.55	1.174–2.060
LAIR-2 protein	3.32	0.69	** < 0.0001**	27.85	7.142–108.62
**Multivariate analysis**					
LncRNA-PAX8-AS1	− 1.86	0.73	**0.01**	0.155	0.037–0.65
LAIR-2 mRNA	0.38	0.17	**0.03**	1.46	1.04–2.06
LAIR-2 protein	4.49	1.02	** < 0.0001**	88.8	11.97–659.2
Constant	− 12.955				

Subclinical hypothyroidism (n = 110) versus healthy controls (n = 95). Log likelihood of the stepwise multivariate logistic regression model =  − 32.015, − 2 Log likelihood = 64.03, *P* < 0.0001. *P* values in bold means statistical significance between groups (*P* < 0.05).^a^ adjusted for age and sex.

## Discussion

Recently, particular attention is paid to discover new biomarkers for hypothyroidism to reinforce its diagnosis and screening. Furthermore, hormonal replacement over-treatment of hypothyroidism was reported to increase the risk of cardiac morbidity and mortality, osteoporosis, cognitive dysfunction, and reduced muscle mass; for these reasons novel biomarkers are needed to evolve the therapy^3,24–26^. Dysfunctional regulation of LncRNAs and mRNAs has been suggested as a novel clue in understanding the pathology of hypothyroidism and thus targeting therapy. However, studies on the impact of *LncRNA-PAX8-AS1* and *LAIR-2* gene polymorphisms with differential expression levels on hypothyroidism are still lacking.

The current study revealed associations of *LncRNA-PAX8-AS1* rs4848320 and rs1110839 with susceptibility to clinical hypothyroidism in our patients, but these SNPs were not associated with subclinical hypothyroidism risk. The study also highlighted the association of *LAIR-2* rs2287828 with the susceptibility to clinical and subclinical hypothyroidism. Interestingly, the haplotype analysis identified that the joint effect of these three variants in the studied population was associated with the increased risk of hypothyroidism in all studied patients. This may be interpreted by that patients who have the T allele for the three variants are more prone to hypothyroidism. Moreover, *LncRNA-PAX8-AS1* rs4848320 and rs1110839 as well as *LAIR-2* rs2287828 were functionally correlated with the serum expression levels of LncRNA-PAX8-AS1 and LAIR-2 mRNA along with its protein, respectively in hypothyroidism. These findings may contribute to the pathogenesis of hypothyroidism, and these SNPs could be used as functional genetic susceptibility markers for sporadic hypothyroidism via functional modulation of LncRNA-PAX8-AS1 and LAIR-2 expression pattern.

To our knowledge, this is the first research to investigate the primary proof of association between *LncRNA-PAX8-AS1* rs4848320 and rs1110839 as well as *LAIR-2* rs2287828 with the increased risk of occurrence and progression of hypothyroidism. The data revealed that the T allele carriers in *LncRNA-PAX8-AS1* rs4848320 and rs1110839 confer high risk against clinical hypothyroidism, but not subclinical hypothyroidism. On the other hand, those carrying the T allele in *LAIR-2* rs2287828 deliberate high risk against both clinical and subclinical hypothyroidism in this study. Surprisingly, the literature on *LncRNA-PAX8-AS1* rs4848320 and rs1110839 showed a variety of approaches; in a Southeast Iranian population, the T allele carriers in *LncRNA-PAX8-AS1* rs4848320 increased the susceptibility of developing childhood acute lymphocytic leukemia, while rs1110839 variant has no effect^19^. In the Han Chinese population, T and G allele carriers in *LncRNA-PAX8-AS1* rs4848320 and rs1110839, respectively, decreased the susceptibility of cervical cancer^14^. Likewise, the T allele carriers in *LAIR-2* rs2287828 were associated with the risk of Pemphigus foliaceus disease in Brazilian population^22^. However, the mechanistic role of these SNPs was not tackled by these studies. Herein, we found that the studied SNPs affected the differential gene expression of the circulating LncRNA-PAX8-AS1 and LAIR-2 mRNA coupled with its protein in hypothyroidism.

This study identified that serum LncRNA-PAX8-AS1 downregulation as well as LAIR-2 upregulation could be implicated in hypothyroidism. Also, the multivariate analysis identified serum LncRNA-PAX8-AS1 and LAIR-2 mRNA levels to be independent predictors for being diagnosed with clinical or subclinical hypothyroidism. Serum LAIR-2 protein was an additional predictor of the diagnosis of subclinical hypothyroidism.

Indeed, LncRNA-PAX8-AS1 was demonstrated as a potential regulator of PAX8, a transcription factor which is crucial for the organogenesis of the developing thyroid gland^15^. The knockout of *PAX8* gene has been reported to reflect the phenotypes of congenital hypothyroid disorders in human patients, delineating disruptions in thyroid hormone biosynthesis (a condition known as dyshormonogenesis)^27^. Moreover, in adult thyroid, PAX8 transcription factor was regarded as a master regulator of the differentiated thyroid phenotype by overlapping with genomic binding sites such as promoter regions and boundary elements close to the 5'-UTRs of identified genes encoding sodium iodide symporter, thyroid peroxidase, and thyroglobulin, thus activating the expression of these critical proteins for the biosynthesis, storage, and secretion of thyroid hormones; T3 and T4^15,28^. Additionally, the expression level of LncRNA-PAX8-AS1 was previously shown to be significantly associated with overall or recurrence-free survival time in patients with papillary thyroid carcinoma^29^. In this study, we identified that downregulation of serum LncRNA-PAX8-AS1 expression level in clinical and subclinical hypothyroidism may predict the risk and help in the diagnosis of hypothyroidism. The significant lower expression of serum LncRNA-PAX8-AS1 in clinical hypothyroidism than in subclinical hypothyroidism may foretell the progression risk of hypothyroidism. Importantly, the two SNPs; rs4848320 and rs1110839 in *LncRNA-PAX8-AS1* gene were demonstrated as eQTLs of *PAX8*, affecting the expression or function of *LncRNA-PAX8-AS1*, thereby altering the expression of PAX8 that regulates the thyroid gland^12,18^. Notably, the finding that *LncRNA-PAX8-AS1* rs4848320 and rs1110839 were not associated with subclinical hypothyroid patients may justify the normal T3 and T4 levels in these patients. Alternatively, the association of these SNPs with clinical hypothyroid patients could rationalize in part the lower T3 and T4 in these patients than other groups. These findings reinforce that LncRNA-PAX8-AS1 could be implemented in the occurrence and progression of hypothyroidism.

Likewise, LAIR-2 protein, a proinflammatory mediator, that underpins the pathogenesis of autoimmune or inflammatory diseases through regulating the inhibitory potential of the membrane-bound LAIR-1 by competition for collagen ligands, resulting in enhanced activation of immune cells, a hallmark of autoimmune hypothyroid diseases^20,21^. Simone et al.^13^ documented higher serum levels of LAIR-2 protein in patients with autoimmune thyroid diseases than in healthy controls, possibly due to different monoclonal antibodies implemented^13^. The current study showed that LAIR-2 upregulation could predict the risk and help in diagnosis of both clinical and subclinical hypothyroidism.

In clinical hypothyroidism, the expression levels of LncRNA-PAX8-AS1 and LAIR-2 mRNA were markedly different compared to subclinical hypothyroidism and healthy control, indicating their diagnostic value. However, no significant correlation between LncRNA-PAX8-AS1 and LAIR-2 mRNA with the clinical laboratory data was shown, meanwhile, LAIR-2 protein was positively correlated with both LDL-cholesterol and total cholesterol levels. Extensive preclinical studies have formerly revealed the critical roles of the innate and adaptive immune systems in promoting cholesterol-induced atherosclerosis-associated chronic inflammation in arterial blood vessels^30^.

Ample data from previous work^31–36^ revealed that circulating LncRNAs along with other genes could be promising diagnostic and prognostic biomarkers for different clinical situations. In this study, we displayed that serum LncRNA-PAX8-AS1, LAIR-2 mRNA and its protein distinguished all hypothyroid patients from healthy controls with moderate to high sensitivity and specificity, and were distinctively expressed between clinical hypothyroid patients and healthy controls or subclinical hypothyroidism with high accuracy, sensitivity, and specificity, implying that they could be used as potential biomarkers for early and new diagnosis of hypothyroidism. Moreover, LncRNA-PAX8-AS1 and LAIR-2 mRNA were distinctively expressed between clinical versus subclinical hypothyroid patients and discriminated both conditions in ROC analysis, indicating their prognostic potential.

However, the limitations of this study shouldn’t be neglected. The relatively small sample size of the study investigations may limit the interpretation of the results. Selection bias may be evident for patients as we collected the different samples from one hospital. The study results should be interpreted with caution when extended to other populations. As a result, the findings of this study need to be validated on a bigger scale or in a community with a diverse range of racial groups.

## Conclusion

This study is the first to propose the *LncRNA-PAX8-AS1* and *LAIR-2* variants as novel genetic biomarkers of hypothyroidism, which could alter the LncRNA-PAX8-AS1 and LAIR-2 expression. Furthermore, the serum LncRNA-PAX8-AS1 and LAIR-2 mRNA and protein levels have the potential as novel diagnostic and prognostic indicators of hypothyroidism. Our findings could be implemented in hypothyroidism screening, genetic treatment, and have the possibility of large-scale application. Finally, the relationship between the studied parameters and the environmental risk factors of hypothyroidism should be investigated.

## Subjects and methods

### Subjects

This study included 305 participants classified as 95 healthy controls and 210 hypothyroid patients recruited from the Internal Medicine Department and Outpatient Endocrine Clinic, Kasr Al-Ainy Hospital, Cairo University from June 2020 to June 2021. Patients were classified into 100 clinical hypothyroid cases and 110 cases with subclinical hypothyroidism depending on serum levels of TSH, free T4, and free T3 and the diagnosis was affirmed by clinical examinations.

Full history taking and clinical assessment were done for all recruited patients. Thyroid function tests were carried out, including serum levels of TSH, free T4, free T3 as well as thyroid volume. Furthermore, the lipid profile parameters [total cholesterol, LDL-cholesterol, HDL-cholesterol and triglycerides] and the glycemic control status of patients [fasting plasma glucose, 2HPP, fasting plasma insulin, and blood HbA1c] were assessed.

Patients whose age above 18 years old from both sexes were included in the study, while those having any current malignancies, severe liver and kidney diseases, other autoimmune diseases (except autoimmune hypothyroidism), active infection, and on any medications including glucocorticoids, hormones and drugs that interfere with thyroid function were excluded.

Clinical hypothyroid patients (92 females/8 males, age range 24–59 years) exhibited elevated serum TSH with low serum free T4 and free T3 levels. Subclinical hypothyroid patients (104 females/6 males, age range 25–58 years) had elevated serum TSH along with normal free thyroid hormones levels. The healthy control group was age- and sex-matched to the patient groups.

The sample size was computed with a power of 0.8 as previously described by Hong and Park, 2012^37^ using the additive model while considering the following assumptions: case–control ratio of 1:1; 5% error rate in an allelic test; MAF; odds ratio of 2; complete linkage disequilibrium; and the prevalence of disease from published data in Egypt^38^.

All participants signed an informed consent. The Research and Ethics Committee for Experimental and Clinical Studies at Faculty of Pharmacy, Cairo University, Egypt approved the study protocol and informed consent (approval number: BC29150) which conformed to the ethical guidelines of Helsinki Declaration.

### Sample collection and storage

Six milliliter blood samples were taken from each participant and separated into two vacutainers. The first 3 mL of blood were collected into EDTA vacutainer tubes for DNA extraction and genotyping and were stored at − 80 °C until used. The other 3 mL of the blood were stored in yellow gel vacutainer tubes, kept at room temperature for 30 min then centrifuged at 4000 rpm for 10 min to detach the sera from the clotted whole blood. The first aliquoted sera were used for RNA extraction, whereas the other aliquoted sera were used for the LAIR-2 protein assay. Until used, all serum aliquots were maintained frozen at a temperature of − 80 °C.

### DNA extraction and genotyping

Genomic DNA was deduced from whole EDTA blood samples of all subjects using QIAamp DNA MiniKit as depicted in the manufacturer's instructions (Qiagen, Valenica, CA). The yield concentration and purity were determined by NanoDrop2000 (ThermoFisher Scientific,Waltham, MA, USA). Pre-designed primer/probe sets for the *LncRNA-PAX8-AS1* SNPs: rs4848320 (C/T) [Assay ID: C_1940037_20, Catalog number: 4351379] and rs1110839 (G/T) [Assay ID: C_1940012_20, Catalog number: 4351379], and the *LAIR-2* SNP rs2287828 (C/T) [Assay ID: C_16183138_20, Catalog number: 4351379] (Applied Biosystems) were used for genotyping using the TaqMan allelic discrimination assay. DNA amplification was done using 12.5 μL TaqMan master mix, 1.25 μL primers/probes, 1 μL DNA, and 10.25 μL H_2_O in a final volume of 25 μL. The real-time PCR was conducted using the Rotor gene Q system (Qiagen) under the following cycling conditions: 95 °C for 10 min, followed by 40 cycles at 92 °C for 15 s and 60 °C for 90 s. At the end point of each cycle, fluorescence was assessed.

### Assay of serum LncRNA-PAX8-AS1 and LAIR 2 mRNA by reverse transcriptase-quantitative polymerase chain reaction (RT-qPCR)

Two hundred microliters of serum were used for total RNA extraction using the miRNeasy extraction kit (Qiagen, Valenica, CA) with the supplied QIAzol lysis reagent as instructed by the manufacturer. The extracted RNA was used for both lncRNA and mRNA expression analysis after evaluation of the concentration and purity using the NanoDrop 2000 model (ThermoFisher Scientific, USA).

Reverse transcription (RT) was carried out on 0.1 μg of total RNA in a final volume 20 μL RT reactions using RT^2^ First Strand kit as recommended by the manufacturer (Qiagen, Valenica, CA). Dilution of the RT product was performed with 50 μL of RNase-free water and the diluted cDNA was stored at − 20 °C until analysis. The Maxima SYBR Green PCR kit (Thermo Fisher Scientific, Waltham, MA, USA) was used in qPCR along with GAPDH as a housekeeping gene with ready-made primers (Qiagen Valenica, CA) for human LncRNA-PAX8-AS1 (Catalog No: 330701, specific number: LPH21217A-200), LAIR-2 mRNA (Catalog No: 249900, specific number: QT00009499) and GAPDH (Catalog No: 249900, specific number: QT00079247). In brief, 2.5 μL appropriately diluted cDNA template was mixed with 5.5 μL RNase free water, 10 μL Maxima SYBR Green PCR Master Mix and 2 μL ready-made forward and reverse primers in a final 20 μL reaction mixture. RT-qPCR was conducted by applying the following conditions: 95 °C for 10 min followed by 40 cycles of 15 s at 95 °C and 60 s at 60 °C using a Rotor Gene Q system (Qiagen).

A melting curve analysis was performed to ensure the specificity of PCR products of the studied genes. 2^−ΔCt^ was employed to compute gene expression relative to the internal control and 2^−ΔΔCt^ was used to calculate the fold change relative to the healthy control group.

### Assessment of serum LAIR-2 protein

A human LAIR-2 ELISA kit (E2299Hu) was obtained from Bioassay Technology Laboratory (Shanghai, China).for the quantitative measurement of serum LAIR-2 protien levels.

### Statistical analysis

SPSS software (Chicago, IL, USA) version 25 and GraphPad Prism 8.0 statistical software (San Diego, CA, USA) were used for data analysis. Data are presented as the median (25%-75% percentiles), mean ± standard deviation (SD) or number (percentage) when appropriate. Kolmogorov–Smirnov and Shapiro–Wilk tests were used for testing normality. Normally distributed data were analyzed by unpaired student t test or one way ANOVA followed by Tukey’s post-hoc test when appropriate. The Mann–Whitney U test or Kruskal–Wallis test followed by Dunn’s post-hoc test were applied to analyze the non-normally distributed data when appropriate. Chi square or Fisher’s exact test were applied to compare the categorical data. The diagnostic and prognostic accuracy of the studied parameters were computed from the ROC curve analysis which calculates the AUC. When AUC is between 0.7 and 0.89, a potential or promising discriminator is considered, if AUC ≥ 0.9 an excellent discriminator is considered. The correlation between the measured parameters was evaluated using Spearman's rho coefficient. To find predictor variables linked to the risk of being diagnosed with clinical or subclinical hypothyroidism, univariate and multivariate logistic regression analyses were conducted. Significant predictor variables from the univariate analysis were incorporated in a stepwise forward multivariate analysis (*P* < 0.05 for inclusion and *P* < 0.1 for exclusion from the model). Age and sex were included as covariates to adjust the data for confounding factors.

SNP analysis and the association of SNPs with clinical and subclinical hypothyroid risk were tested using logistic regression models controlling for age and sex by employing the SNPStats online software (InistitutCatalà d’Oncologia, Barcelona, Spain; https://www.snpstats.net/start.htm). The reference category was set as the major allele or the major homozygote genotype in the control population. The best fit model of each SNP was chosen when it has the lowest Akaike information criterion (AIC) and the Bayesian information criterion (BIC) values compared to other genetic models. Statistical significance was set as *P* < 0.05 for all tests, with a 95% CI.

### Ethics approval and consent to participate

All participants signed an informed consent. The Research and Ethics Committee for Experimental and Clinical Studies at Faculty of Pharmacy, Cairo University, Egypt approved the study protocol and informed consent (approval number: BC29150) which conformed to the ethical guidelines of Helsinki Declaration.

## Supplementary Information

Supplementary Information.

## Data Availability

All data generated or analyzed during this study are included in this published article and its supplementary information files.
