# Supplementary material for: Association of LncRNA-PAX8-AS1 and LAIR-2 polymorphisms along with their expression with clinical and subclinical hypothyroidism
Source: Sci Rep. 2023 Jan 2;13:6. doi: 10.1038/s41598-022-26346-0 (PMC9807632; doi:10.1038/s41598-022-26346-0)
Supplement: Supplementary file 1 — Supplementary Information. [file 41598_2022_26346_MOESM1_ESM.docx]

**Supplementary Table S1 Hardy‐Weinberg equilibrium for studied SNPs**

| **Hardy-Weinberg equilibrium** | | | | | | | | |
| --- | --- | --- | --- | --- | --- | --- | --- | --- |
| ***LncRNA-PAX8-AS1* rs4848320** | | | | | | | | |
| **Control** | | | | **Clinical hypothyroidism** | | | | |
| ***P*=0.29** | TT | CT | CC | ***P*=0.16** | TT | CT | CC | **Genotype** |
|  | 4 | 42 | 49 |  | 14 | 56 | 30 | **Observed** |
| **Control** | | | | **Subclinical hypothyroidism** | | | | |
|  |  |  |  | ***P*=0.51** | TT | CT | CC | **Genotype** |
|  |  |  |  |  | 12 | 44 | 54 | **Observed** |
| ***LncRNA-PAX8-AS1* rs1110839** | | | | | | | | |
| **Control** | | | | **Clinical hypothyroidism** | | | | |
| ***P*=0.23** | TT | GT | GG | ***P*=0.23** | TT | GT | GG | **Genotype** |
|  | 6 | 46 | 43 |  | 21 | 57 | 22 | **Observed** |
|  |  |  |  | **Subclinical hypothyroidism** | | | | |
|  |  |  |  | ***P*=0.18** | TT | GT | GG | **Genotype** |
|  |  |  |  |  | 14 | 41 | 55 | **Observed** |
| ***LAIR 2* rs2287828** | | | | | | | | |
| **Control** | | | | **Clinical hypothyroidism** | | | | |
| ***P*=0.095** | TT | CT | CC | ***P*=0.41** | TT | CT | CC | **Genotype** |
|  | 3 | 15 | 77 |  | 16 | 54 | 30 | **Observed** |
|  |  |  |  | **Subclinical hypothyroidism** | | | | |
|  |  |  |  | ***P*=0.53** | TT | CT | CC | **Genotype** |
|  |  |  |  |  | 16 | 47 | 47 | **Observed** |

Hardy‐Weinberg equilibrium was done by chi‐square test. *P*<0.05 means statistical significance.

**Supplementary Table S2 Stratification analysis of the effect of rs4848320 (C/T), rs1110839 (G/T) and rs2287828 (C/T) SNPs on clinical and subclinical hypothyroid risk by gender.**

| ***Clinical hypothyroid risk*** | | | | | | | | | | | |
| --- | --- | --- | --- | --- | --- | --- | --- | --- | --- | --- | --- |
| **Parameter** | ***rs4848320 (C/T)*** | | *P*^a^, OR^a^ (95%CI) | ***rs1110839 (G/T)*** | | | | ***P*^a^, OR^a^ (95%CI)** | ***rs2287828 (C/T)*** | | ***P*^a^, OR^a^ (95%CI)** |
|  | ***Model*** | |  | ***Model*** | | | |  | ***Model*** | |  |
| **Female** | Log additive | | **< 0.0001,** 2.94 (1.73-4.99) | Log additive | | | | **< 0.0001,** 2.51 (1.52-4.14) | Dominant | | **< 0.0001,** 21.61 (9.58-48.75) |
|  |  |  |  |  |  |  |  |  | CT+TT | CC |  |
|  | ------------------ | |  | ------------------ | | | |  | (68/10) | (24/77) |  |
| **Male** | Log additive | | 0.06, 0.1 (0.01-1.09) | Recessive | | | | 0.082 | Recessive | | 0.24 |
|  |  |  |  | TT | GG+GT | | |  | TT | CC+CT |  |
|  | ------------------ | |  | (2/0) | (6/8) | | |  | (0/1) | (8/7) |  |
| ***Subclinical hypothyroid risk*** | | | | | | | | | | | |
| **Parameter** | ***rs4848320 (C/T)*** | | ***P*^a^**, **OR^a^ (95%CI)** | ***rs1110839 (G/T)*** | | | | ***P*^a^, OR^a^ (95%CI)** | ***rs2287828 (C/T)*** | | ***P*^a^, OR^a^ (95%CI)** |
|  | ***Model*** | |  | ***Model*** | | | |  | ***Model*** | |  |
| **Female** | Recessive | | 0.19, 2.37 (0.61-9.21) | Recessive | | | | 0.14, 2.1 (0.77-5.73) | Dominant | | **< 0.0001,** 12.77 (5.80-28.14) |
|  | TT | CC+CT |  | TT | | GG/GT | |  | CT+TT | CC |  |
|  | (9/3) | (95/84) |  | (14/6) | | (90/81) | |  | (62/10) | (42/77) |  |
| **Male** | Log additive | | 0.05, 0.13 (0.01-1.45) | GT against GG | | | | 0.14, 0.14 (0.01-2.54) | Recessive | | 0.42 |
|  |  |  |  | GT | | | GG |  | TT | CC+CT |  |
|  | ------------------ | |  | (1/4) | (5/4) | | |  | (0/1) | (6/7) |  |

The best fit model is demonstrated for each SNP chosen using the lowest Akaike information criterion (AIC) and the Bayesian information criterion (BIC) values compared to other genetic models. OR: Odds ratio, ^a^adjusted for age in a logistic regression model using SNPstats online software. Values in brackets are expressed as numbers (cases/controls). *P* values in bold are statistically significant (*P* < 0.05)

**Supplementary Table S3 Correlation analysis**

|  |
| --- |

| **LAIR 2 protein** | **LAIR 2 mRNA** | **LncRNA-PAX8-AS1** |  | **Parameters** |
| --- | --- | --- | --- | --- |
| -0.13 | 0.19 | ― | **r** | **LncRNA-PAX8-AS1** |
| 0.22 | 0.06 | ― | ***P*** |  |
| -0.04 | ― | 0.19 | **r** | **LAIR 2 mRNA** |
| 0.71 | ― | 0.06 | ***P*** |  |
| ― | -0.04 | -0.13 | **r** | **LAIR 2 protein** |
| ― | 0.71 | 0.22 | ***P*** |  |
| -0.13 | 0.07 | -0.07 | **r** | **Age** |
| 0.19 | 0.51 | 0.51 | ***P*** |  |
| 0.03 | 0.04 | -0.02 | **r** | **Weight (kg)** |
| 0.74 | 0.67 | 0.88 | ***P*** |  |
| 0.14 | 0.08 | 0.02 | **r** | **BMI (Kg/m2)** |
| 0.16 | 0.42 | 0.84 | ***P*** |  |
| 0.17 | -0.07 | -0.05 | **r** | **TSH (mIU/L)** |
| 0.09 | 0.52 | 0.64 | ***P*** |  |
| -0.16 | 0.07 | 0.07 | **r** | **Free T4 (ng/dL)** |
| 0.12 | 0.49 | 0.49 | ***P*** |  |
| 0.02 | 0.14 | 0.13 | **r** | **Free T3 (pg/dL)** |
| 0.85 | 0.16 | 0.19 | ***P*** |  |
| -0.08 | -0.04 | 0.17 | **r** | **Thyroid Volume (mL)** |
| 0.44 | 0.66 | 0.09 | ***P*** |  |
| 0.21 | 0.04 | -0.01 | **r** | **Total cholesterol (mg/dL)** |
| **0.04** | 0.66 | 0.89 | ***P*** |  |
| 0.28 | -0.01 | -0.07 | **r** | **LDL-cholesterol (mg/dL)** |
| **0.004** | 0.90 | 0.49 | ***P*** |  |
| -0.10 | 0.18 | -0.09 | **r** | **HDL-cholesterol (mg/dL)** |
| 0.36 | 0.07 | 0.36 | ***P*** |  |
| -0.17 | 0.02 | 0.09 | **r** | **Triglycerides (mg/dL)** |
| 0.10 | 0.85 | 0.40 | ***P*** |  |
| 0.01 | -0.02 | -0.19 | **r** | **FBG (mg/dL)** |
| 0.93 | 0.87 | 0.06 | ***P*** |  |
| -0.05 | -0.08 | -0.18 | **r** | **2HPP blood glucose (mg/dL)** |
| 0.63 | 0.46 | 0.08 | ***P*** |  |
| 0.07 | 0.08 | -0.12 | **r** | **HBA1C (%)** |
| 0.49 | 0.44 | 0.26 | ***P*** |  |
| -0.17 | -0.08 | -0.18 | **r** | **Fasting insulin** |
| 0.10 | 0.44 | 0.07 | ***P*** |  |

Correlations were conducted using Spearman correlation. r, Spearman rho coefficient. *P* values in bold means statistical significance (*P*< 0.05).

**Supplementary Figure S1. Association of rs4848320 genotypes with clinicopathological data of clinical hypothyroid patients**. In these figures (A-M), non-filled bars represent the median and interquartile range of non-normally distributed data analyzed by test Mann-Whitney U or Kruskal-Wallis followed by Dunn’s tests when appropriate, while filled bars represent mean ± SD of normally distributed data analyzed by unpaired student t test or ANOVA followed by Tukey’s test when appropriate. *P*<0.05 was considered statistically significant.

**Supplementary Figure S2. Association of rs1110839 genotypes with clinicopathological data of clinical hypothyroid patients**. In these figures (A-M), non-filled bars represent the median and interquartile range of non-normally distributed data analyzed by test Mann-Whitney U or Kruskal-Wallis followed by Dunn’s tests when appropriate, while filled bars represent mean ±SD of normally distributed data analyzed by unpaired student t test or ANOVA followed by Tukey’s test when appropriate. *P*<0.05 indicates statistical significance.

**Supplementary Figure S3. Association of rs2287828 genotypes with clinicopathological data of clinical hypothyroid patients**. In these figures (A-M), non-filled bars represent the median and interquartile range of non-normally distributed data analyzed by test Mann-Whitney U or Kruskal-Wallis followed by Dunn’s tests when appropriate, while filled bars represent mean±SD of normally distributed data analyzed by unpaired student t test or ANOVA followed by Tukey’s test when appropriate. *P*<0.05 indicates statistical significance.
